# The Performance of Ti/Steel Joints Welded by Resistance Spot Welding with a Nickel Interlayer

**DOI:** 10.3390/ma18143247

**Published:** 2025-07-10

**Authors:** Nannan Wang, Gang Li, Yanling Hu, Hongxin Shi, Ranfeng Qiu, Keke Zhang

**Affiliations:** 1 School of Materials Science and Engineering, Henan University of Science and Technology, Luoyang 471023, China; 15896555218@163.com (N.W.); 18939450281@163.com (G.L.); hyhx66@163.com (H.S.); zhkekekd@163.com (K.Z.); 2 Luoyang College, Civil Aviation Flight University of China, Luoyang 471000, China; 3 State Key Laboratory of High Temperature Light Alloys and Application Technology, Luoyang 471039, China; 4 Science and Technology Innovation Center of Intelligent Equipment & Advanced Materials, Longmen Laboratory, Luoyang 471039, China; huyanling2023@163.com

**Keywords:** titanium, steel, resistance spot welding, nickel interlayer

## Abstract

Resistance spot welding was performed to join a 2 mm thick TA2 titanium plate and Q235 steel plate using nickel foil with thicknesses of 0.02 mm, 0.04 mm, and 0.06 mm as interlayers. The microstructure of the nugget zone and the interface region of the joint were systematically observed and analyzed, and the tensile shear-bearing capacity of the joint was evaluated. As the welding current increased, the tensile shear load of the joint exhibited a trend of initially increasing and subsequently decreasing. When the welding current was 8 kA, the tensile shear load of the joints with an interlayer of 0.04 mm thickness reached a maximum value of 8.02 kN. The results indicate that employing a reduced welding current can effectively prevent the mixing of nuggets on both sides of the titanium and steel interface. This ensures that the intermetallic compounds formed in the interface region are confined to the Ti-Ni series, which is crucial for enhancing the tensile shear load of the joint.

## 1. Introduction

Titanium and its alloys have gained extensive applications in aerospace, shipping, petrochemicals, the automotive industry, nuclear power, and other fields owing to their exceptional properties, including high specific strength, superior corrosion resistance, remarkable high-temperature strength, and excellent low-temperature toughness [1]. However, their application is somewhat limited owing to their relatively high cost, low elastic modulus, and insufficient creep resistance. Dissimilar material structural components can fully leverage the respective advantages of these two materials, create complementary strengths, and thus hold broad application prospects. The composite structure of titanium and steel with high strength and toughness offers advantages such as lightweight design, low cost, and excellent corrosion resistance, which holds great significance for further expanding the application scope of titanium and its alloys [2]. However, achieving this necessitates the effective joining of titanium and steel, which presents a significant challenge due to the substantial differences in their physical and metallurgical properties.

A substantial quantity of hard and brittle intermetallic compounds (IMCs) formed at the joining interface when titanium and steel were directly welded using the fusion welding process, which significantly compromises the mechanical properties and reliability of the joint [3]. To suppress the growth of IMCs and mitigate their adverse effects on joint performance, specific solid-state welding techniques, which are characterized by relatively low welding temperatures, such as explosion welding [4], diffusion bonding [5], ultrasonic welding [6], and friction welding [7], were employed for joining titanium and steel. Although prior studies have demonstrated that solid-state welding technology yields promising results in titanium/steel welding [1], each welding technique exhibits specific characteristics and inherent limitations when applied in practice [8]. Therefore, in addition to solid-state welding, it is also necessary to explore the feasibility of joining titanium and steel using other welding techniques. Resistance spot welding (RSW) is a welding technique specifically designed for joining thin plates in lap configurations. While it is classified as a pressure welding process, localized melting of the base metal occurs during the welding operation. Therefore, directly joining titanium and steel through RSW poses significant challenges due to the formation of a substantial number of IMCs at the welding interface [9].

For the welding of dissimilar materials, the application of an interlayer (or filler material) can effectively suppress the formation of brittle IMCs or promote the generation of less brittle IMCs as replacements, which is widely recognized as a robust strategy to enhance the mechanical and metallurgical performance of the joint [10]. Unfortunately, the addition of a single pure metal interlayer (or filler material) is insufficient to overcome the brittleness of Ti/steel joints [2]. This suggests that suitable interlayers must be carefully selected based on the characteristics of the base materials on both sides and used in combination to achieve optimal performance. In prior research [11], considering the properties of titanium, a niobium foil was chosen as an interlayer to conduct RSW between Ti and steel. It was observed that the Nb interlayer achieved a strong bond with the titanium base material, while a FeNb layer formed at the Nb/steel interface within the joint [11]. In this study, an interlayer was selected according to the specific characteristics of steel for the RSW of Ti/steel with the aim of providing fundamental insights for subsequent research on the RSW of Ti/steel with bimetallic interlayers.

Among the commonly used metals, Cu and Ni exhibit relatively high solid solubility in iron (the primary component of steel) and are thus considered suitable interlayer materials for Ti/steel welding [10]. However, in comparison to Cu, Ni has a linear expansion coefficient that is closer to that of the base materials, thereby reducing the risk of thermal stress-induced damage during welding [12]. In the investigation of other welding techniques for Ti/steel using Ni as an interlayer, it was observed that Ti-Ni IMCs formed in the interface region [13,14,15]. These Ti-Ni IMCs exhibited higher ductility compared to Ti-Fe IMCs, thereby improving the performance of the joint [13,14,15]. Therefore, Ni was chosen as the interlayer material for the RSW of Ti/steel, and a comprehensive investigation was carried out into the microstructure and mechanical properties of the joint.

## 2. Experimental Materials and Procedures

In this study, the base materials consisted of a 2 mm thick TA2 pure titanium plate and a Q235 steel plate, both of which were machined into dimensions of 100 mm × 30 mm. Their chemical composition is listed in Table 1. A 0.02 mm thick pure nickel foil with shapes of 30 mm × 30 mm in size was used as the interlayer. The nickel interlayer used for welding was available in three different thicknesses: 0.02 mm (1 layer), 0.04 mm (2 layers), and 0.06 mm (3 layers). Before welding, the oxides on the surfaces of the titanium plates and Q235 steel plates were removed using a wire brush. Subsequently, the surfaces were cleaned with acetone and dried with hot air to ensure adequate preparation for welding. The titanium plate and Q235 steel plate, after undergoing cleaning treatment, were overlapped and assembled along the length direction with a lap length of 30 mm. Meanwhile, a Ni foil interlayer was inserted between the titanium plate and the Q235 steel plate.

The titanium plate was positioned on the upper electrode side and was welded using a medium-frequency variable-frequency RSW machine (DM200, Medar, Shanghai, China). The electrode employed had a tip diameter of 6 mm and was fabricated from a CrZrCu alloy. The flow rate of cooling water passing through the electrodes was 30 L per minute. The RSW was performed by varying the welding current and welding time, respectively. The detailed welding conditions are presented in Table 2. Here, the selection of the welding parameter range was guided by findings from a previous relevant study [11]. Under each set of combined welding parameter conditions, seven specimens were fabricated, two of which were allocated for the microstructure observation of the joint, while the remaining five were utilized for tensile testing.

After welding, the tensile shear test of the joint was carried out at room temperature using the WDW-100D microcomputer-controlled electronic universal testing machine (AG-1205 kN, Shimadzu, Kyoto, Japan) at a rate of 1 mm/min. The joint was cut along the diameter of the weld, and its cross-section was ground and polished. The microstructure of the joint was observed using a scanning electron microscope (SEM, JSM-6300, JEOL, Tokyo, Japan) equipped with an energy-dispersive spectrometer (EDS, EDAX, Phoenix, AZ, USA), and the composition of the characteristic zones was analyzed. The phase composition was analyzed by X-ray diffraction (XRD, Bruker, Karlsruhe, Germany) in the cross-section and fracture of the joint.

## 3. Results and Discussion

**Figure 1:**
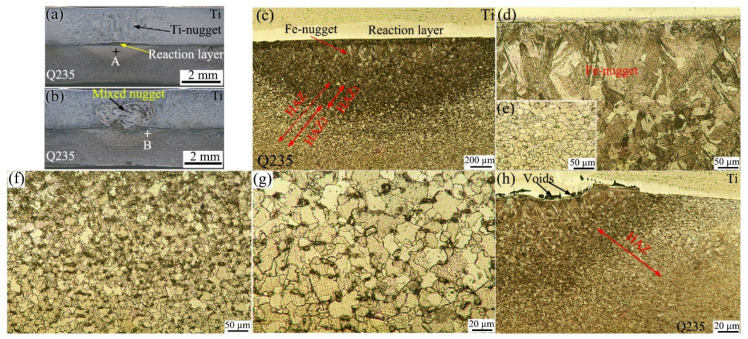
Cross-sections and metallograph of Ti/steel joint: (**a**) cross-section (7 kA); (**b**) cross-section (9 kA); (**c**) enlarged view at location A; (**d**) Fe-nugget; (**e**) base metal of steel; (**f**) HAZ_1_; (**g**) HAZ_2_; and (**h**) enlarged view at location B.

Figure 1a,b present the cross-sections of the joints between TA2 pure titanium and Q235 steel (hereafter referred to as Ti/steel joints), which were produced by RSW with a 0.04 mm thick nickel foil interlayer under various currents of 7 kA and 9 kA, respectively. As shown in Figure 1a, a nugget was observed on the Ti side (hereafter referred to as the Ti-nugget), which consisted of coarse grains. On the steel side, a nearly semi-circular light gray area was observed (taken from location A in Figure 1a), and its enlarged view is presented in Figure 1c. As shown, a nugget was also formed on the steel side (hereafter referred to as the Fe-nugget). Figure 1d presents an enlarged view of the local nugget, clearly demonstrating that the grain size within the Fe-nugget is substantially larger than that in the base steel shown in Figure 1e. As illustrated in Figure 1c, the heat-affected zone (HAZ), approximately 750 µm in width and formed outside the Fe-nugget, can be subdivided into two distinct regions: the dark area (HAZ_1_) adjacent to the nugget and the light area (HAZ_2_) near the base material. Figure 1f,g present the magnified images of the local regions for HAZ_1_ and HAZ_2_, respectively. As shown, the grain size in the HAZ was relatively uneven, primarily consisting of ferrite and pearlite. This is because, upon being heated at a specific temperature, some grains underwent phase transformation and formed austenite. Subsequently, at a higher cooling rate, these grains transformed into fine ferrite and pearlite. Meanwhile, the ferrite grains that did not austenitize during the heating process coarsened due to grain growth. Compared with HAZ_2_, more grains in HAZ_1_ underwent phase transformation following austenitization.

**Figure 2:**
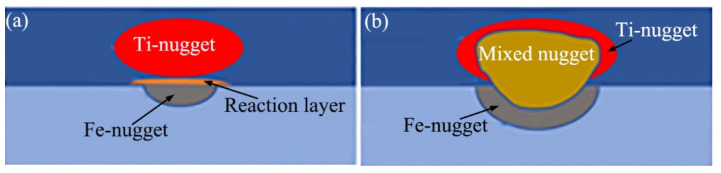
Schematic diagram of the Ti/steel joint; (**a**) joint with DS-nugget; and (**b**) joint with C-nugget.

As depicted in Figure 1a,c, a reaction layer formed between the Ti-nugget and the Fe-nugget, effectively isolating the two. Therefore, this type of joint is referred to as the joint with a double-single nugget (DS-nugget), and its structure is illustrated in Figure 2a. As the welding current increased, a greater amount of metal melted during the welding process. The reaction layer was breached, allowing the nuggets on both sides to penetrate and form a mixed nugget, as shown in Figure 1b. This type of joint is referred to as a joint with a collusion nugget (C-nugget), and its structure is illustrated in Figure 2b. In cases with an interlayer at thicknesses of 0.02 mm, 0.04 mm, and 0.06 mm, when the welding current exceeded 8 kA, the obtained joints were the joint with the C-nugget in this study. This welding current level is referred to as the critical welding current. Figure 1h shows an enlarged view of location B in Figure 1b. As shown, the microstructure of the Fe-nugget and the HAZ outside it on the steel side of the joint with the C-nugget was similar to that observed in the joint with the DS-nugget. In addition, some voids were observed between the Fe-nugget and the mixed nugget. This phenomenon can be attributed to the formation of IMCs within the mixed nugget, as detailed later, which induced volume shrinkage during solidification.

**Figure 3:**
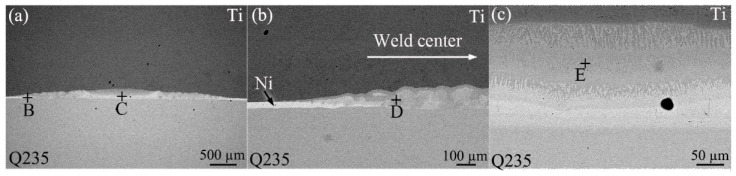
SEM images of the Ti/steel joint with DS-nugget: (**a**) an interface SEM image; (**b**) enlarged image at location B; and (**c**) enlarged image at location C.

Figure 3a presents an SEM image of the interfacial zone between Ti and steel in the joint with the DS-nugget. As shown, layered structures were observed at the interface between Ti and steel. Figure 3b,c show the enlarged images of the peripheral zone (location B) and the central zone (location C) of the weld, respectively. In the peripheral zone of the weld, the Ni interlayer was observed, effectively bonding the Ti and steel on both sides, as illustrated in Figure 3b. When approaching the weld center, the Ni interlayer gradually disappeared, and layered reactants were formed. In the central zone of the weld, layers of dendritic reaction products were formed near the Ti side and the steel side, respectively, as illustrated in Figure 3c.

**Figure 4:**
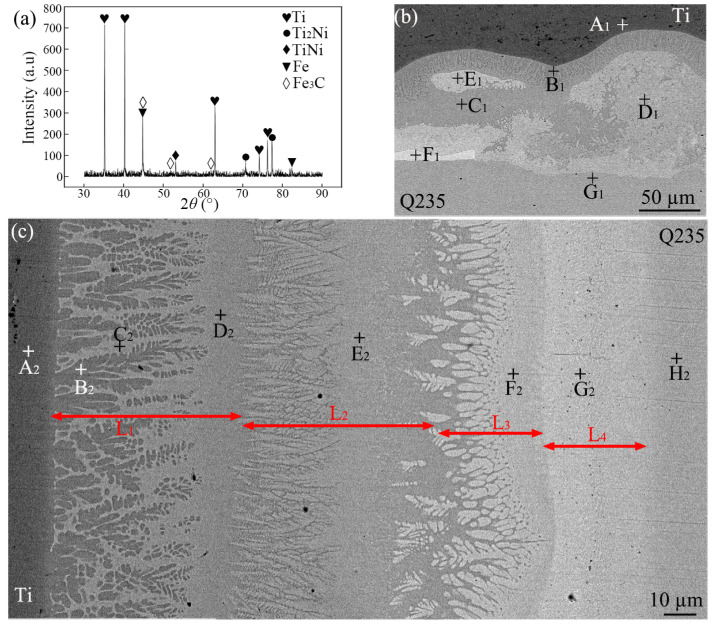
XRD results and SEM images of the Ti/steel joint with the DS-nugget: (**a**) XRD results obtained from the cross-section of the joint; (**b**) an enlarged image at location D in Figure 3; and (**c**) an enlarged image at location E in Figure 3.

Figure 4a presents the XRD analysis results obtained from the cross-section of the joint containing the DS-nugget. In addition to Ti and Fe, the presence of the phases Ti_2_Ni, TiNi, and Fe_3_C was also detected. It can be observed that the peak intensity at 35.093°, corresponding to the (100) crystal plane of Ti, is relatively high. This indicates that the Ti grains exhibit a certain degree of preferred orientation. This phenomenon may occur in the base titanium region surrounding the nugget, which was formed during the rolling process. Figure 4b,c display the high-magnification SEM images at locations D and E in Figure 3, respectively. EDS analysis was performed at the corresponding feature locations, and the results are summarized in Table 3. A small amount of Ni was detected in both the base Ti metal (location A_1_) and the steel (location G_1_), resulting from Ni diffusion to the base metals of both sides during welding. Similarly, Fe and Ti were detected in the residual interlayer (location F_1_). As shown in Figure 4b, within the interfacial region between steel and Ti, two types of reactants were predominantly observed: dark gray reactants (at locations B_1_ and C_1_) and light gray reactants (at locations D_1_ and E_1_). According to the EDS results, the dark gray reactants are inferred to represent a structure composed of (α-Ti) and Ti_2_Ni, while the light gray reactants correspond to the TiNi phase. The observation results indicate that the TiNi phase predominantly forms near the steel side, whereas the Ti_2_Ni phase is primarily located closer to the Ti side. During the heating process, Ni, which has a higher resistivity, heated up more rapidly and exhibited greater reactivity, thereby diffusing quickly toward the base metals on both sides. When the Ti-Ni reached the eutectic composition, liquefaction occurred at 942 °C [16]. As the heating process continued and the temperature increased further, more Ti and Ni transitioned into the liquid phase, while solid-state Ti and Ni also dissolved into liquid metal. At the periphery of the weld, the shorter high-temperature duration led to the inadequate mixing of the liquid phase. During the subsequent cooling and solidification process, as the temperature decreased to 1310 °C, the Ni-rich region experienced a liquid-solid phase transformation of invariant composition, thereby forming TiNi [16]. As the temperature decreased, the Ti-rich region experienced eutectic transformation, leading to the formation of (β-Ti) + Ti_2_Ni. Further reductions in temperature caused the subsequent formation of (α-Ti) and Ti_2_Ni.

Due to the long duration of high temperatures in the center zone of the weld, Ni and Ti atoms diffused fully to the steel side, and Fe atoms diffused into the liquid phase. After solidification, a multi-layer structure, as shown in Figure 4c, was formed. Based on the EDS results, it can be inferred that the L_1_, L_2_, L_3_, and L_4_ layers were primarily composed of (α-Ti) + Ti_2_Ni, TiNi, TiNi + Ti(Ni, Fe)_3_, and (Fe, Ni), respectively. Yu et al. employed MSC Marc software to simulate the temperature field during RSW between a 2 mm thick steel plate and a 2 mm thick titanium alloy plate, using a Ni-Cu alloy as the interlayer [17]. The simulation results indicated that the peak temperature on the steel side was lower than that on the titanium alloy side [17]. During the cooling process, therefore, on the side adjacent to the steel where diffused Ni and Ti atoms were present, an (Fe, Ni) solid solution layer (L_4_ layer) was formed as the temperature decreased. Subsequently, the temperature continued to decrease, and a eutectic reaction occurred at the front of the L_4_ layer, leading to the formation of TiNi and a Ti(Ni, Fe)_3_ eutectic structure layer (L_3_ layer). Here, owing to the relatively scarce formation of Ti(Ni, Fe)_3_, its presence was not detectable by XRD. Then, at the forefront of the L_3_ layer, a solid–liquid phase transformation occurred, resulting in the formation of the TiNi layer (L_2_ layer). Meanwhile, (β-Ti) was initially precipitated near the Ti side, with Ni atoms being expelled to the surrounding regions. As the temperature continued to decrease, a eutectic transformation occurred, resulting in the formation of (β-T) + Ti_2_Ni. Then, β-Ti transformed to (α-Ti) + Ti_2_Ni (eutectoid reaction).

To summarize, although IMCs were formed in the joint with the DS-nugget, the predominant IMCs identified belonged to the Ti-Ni series.

**Figure 5:**
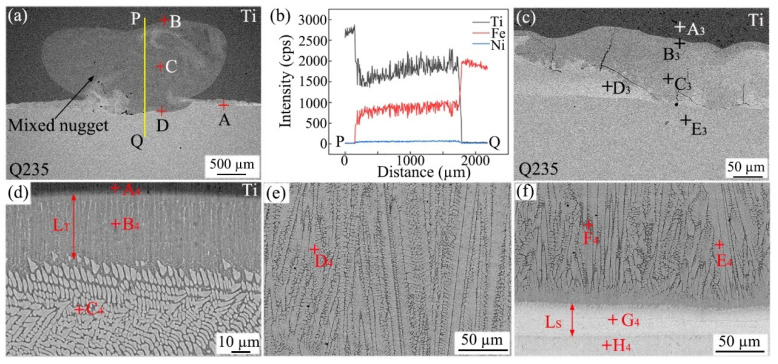
The SEM images and EDS results of the Ti/steel joint with the C-nugget: (**a**) SEM image of the cross-section; (**b**) EDS results; and (**c**–**f**) show an enlarged image taken from A, B, C and, respectively.

Figure 5a shows the SEM image of the cross-section of the Ti/steel joint with the C-nugget. EDS analysis was performed along the PQ line, as depicted in Figure 5a, and the resulting data are shown in Figure 5b. It can be seen from the EDS results that the mixed nugget in the joint primarily consisted of Ti-Fe IMCs. Layered reactions were observed at the Ti/steel interface beside the mixed nugget. Figure 5c presents the SEM image of the Ti/steel interface zone in the joint containing a C-nugget, captured at location A in Figure 5a. EDS analysis was performed at the characteristic locations, and the resulting data are presented in Table 4. According to the EDS results, the microstructure’s composition in the interfacial zone was similar to that of the joint with DS-nugget as both are predominantly composed of Ti-Ni series IMCs. However, the reaction layer formed in the Ti/steel interface zone of the joint containing the C-nugget contained a certain concentration of Fe. This not only increased the brittleness of the reaction layer but also resulted in crack formation, as illustrated in Figure 5c.

Figure 5d–f present the SEM images of the boundaries and central region of the mixed nugget, which were obtained from locations B, C, and D in Figure 5a, respectively. EDS analysis was performed at the characteristic locations, and the resulting data are also presented in Table 4. According to the EDS analysis, it can be inferred that the L_T_ layer adjacent to the Ti side was primarily composed of (α-Ti) and TiFe, whereas the L_S_ layer adjacent to the steel side consisted predominantly of (Fe) and TiFe_2_. The remaining portion of the mixed nugget was composed of coarse dendritic TiFe and interdendritic (α-Ti), resembling the structure of the nugget in the Ti/steel joint directly welded by RSW [18]. The Ti-nugget and the Fe-nugget were mixed to form the mixed nugget in the joint welded under conditions of a high welding current or extended welding time. However, the very brief heating time during the RSW, combined with the closed nature of the nugget and its relatively weak internal stirring force, resulted in only the partial mixing of the nuggets on either side of the welding interface. After the power was cut off, the liquid metal began to solidify from both sides under the cooling influence of the electrodes. Near the Ti side, owing to the lower concentrations of Fe and Ni, the (β-Ti) precipitate phase formed initially during solidification. Subsequently, as the temperature decreased, eutectic transformation occurred, leading to the formation of a (β-Ti) + TiFe eutectic structure. Upon further cooling, the (α-Ti) + TiFe phase (L_T_ layer) was formed. Similarly, an L_S_ layer composed of (Fe) + TiFe_2_ was also formed on the side adjacent to the steel in the meantime. During the subsequent solidification process, owing to the Ti and Fe concentrations being nearly equimolar (approximately 1:1), a significant number of TiFe dendrites formed alongside a minor presence of α-Ti. These phenomena are elaborated upon in reference [18].

**Figure 6:**
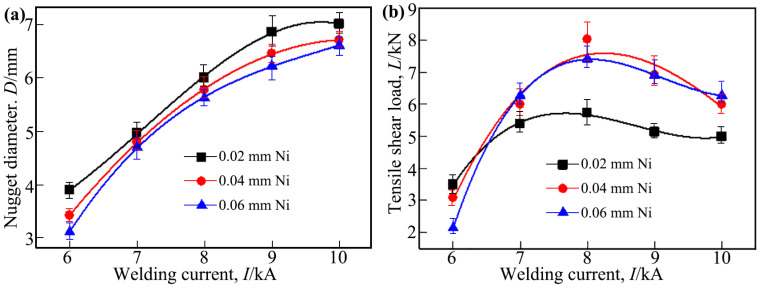
The effect of the welding current on the nugget diameter and tensile shear load of the Ti/steel joint; (**a**) nugget diameter; (**b**) tensile shear load.

Figure 6a,b illustrate the effects of the welding current on the nugget diameter and the tensile shear load of the Ti/steel joint, respectively. Here, the nugget diameter refers to the average measurements taken in mutually perpendicular directions on the Ti side fracture surface of the joint. As shown, with the increase in the welding current, the nugget diameter for joints with all three thicknesses of the interlayer increased accordingly. This is because the increase in the welding current led to an increase in the heat generated during the welding, and thus, more metal melted. Under the same welding current, the nugget diameter of the resulting joint decreased as the thickness of the applied Ni interlayer increased; however, this reduction was not substantial, as shown in Figure 6a. This may be attributed to the relatively high thermal conductivity of Ni, which led to the use of the application of a thicker interlayer, resulting in greater heat loss.

For the three types of joints used, as the welding current increased, the tensile shear load exhibited a trend of initially increasing and subsequently decreasing, as shown in Figure 6b. When the welding current was 8 kA, the tensile shear load of the joints with interlayers of 0.02 mm, 0.04 mm, and 0.06 mm thickness reached their maximum values, which were approximately 5.73 kN, 8.02 kN, and 7.41 kN, respectively.

The joints obtained in this study consistently demonstrated an interface tearing failure mode during the tensile test. For joints with interface tearing failure, the factors influencing the tensile shear load of the joint were mainly the nugget size and the microstructure of the interface zone. As mentioned earlier, within the welding current range of 6 kA to 8 kA, the welded joints featured the DS-nugget and the IMCs formed in these joints belonged to the Ti-Ni series. In contrast, when the welding current was greater than 8 kA, the resulting joints exhibited a C-nugget, with a significant number of Ti-Fe series IMCs forming in the joints. Therefore, as the welding current increased within the 6~8 kA range, the nugget diameter increased, consequently enhancing the tensile shear load of the joint. However, when the welding current was greater than 8 kA, although the nugget diameter also increased with the increase in the welding current, the tensile shear load of the joint actually decreased as the welding current increased. This is because a large number of Ti-Fe series IMCs were formed in the joint in this case, which are more brittle than Ti-Ni series IMCs [13,14,15]. The formation of Ti-Fe series IMCs in the joint also induced the formation of voids and cracks. This led to a decrease in the tensile shear load of the joint under larger welding currents. Furthermore, under the same welding current, the application of an interlayer with a thickness of 0.04 mm or 0.06 mm resulted in no significant difference in the tensile shear load of the joints. However, when an interlayer of 0.02 mm thickness was used and welded with a current exceeding 7 kA, the tensile shear load of the joints was found to be lower. This is attributed to the fact that the main load-bearing area occupied a smaller proportion of the entire joint (as described later) when a thinner interlayer (0.02 mm) and a higher welding current were used.

**Figure 7:**
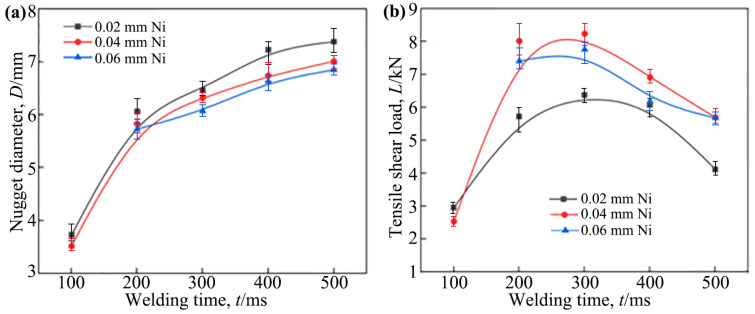
The effect of the welding time on the nugget diameter and tensile shear load of the Ti/steel joint: (**a**) nugget diameter; (**b**) tensile shear load.

Figure 7a,b illustrate the effects of welding time on the nugget diameter and the tensile shear load of the Ti/steel joint, respectively. By comparing Figure 7 with Figure 6, it is evident that the influence of the welding time on the nugget diameter and the tensile shear load of the joint exhibited a similarity to the effect of the welding current on these. For the RSW, the welding time, similar to the welding current, primarily influenced the microstructure and properties of the joint by affecting heat generation and distribution. Therefore, the internal factors responsible for the changes in nugget diameter and tensile shear load, as depicted in Figure 7, with an increase in welding time, are consistent with the previously mentioned analyses. Additionally, it may have been noticed that when the thickness of the interlayer was 0.06 mm and the welding time was 100 ms, data were missing. This was because, in this case, effective joining was not achieved.

It should be noted that the data shown in Figure 6 and Figure 7 are from an average of five samples. The marked deviations represent the maximum and minimum absolute deviations. The test results show that the deviation values of the nugget diameter and tensile shear load were within ±10% of the corresponding average value. 

Direct RSW was performed on a 1 mm thick TA2 plate and a 1 mm thick Q235 steel plate without using an interlayer, with a welding current of 8 kA. The resulting joint exhibited a tensile shear load of approximately 2.9 kN [18]. At a welding current of 8 kA, the maximum tensile shear load for the joint between a 2 mm thick TA2 plate and a 2 mm thick Q235 steel plate, achieved using direct RSW, was approximately 4.8 kN [19]. A 0.1 mm thick Nb foil was used as the interlayer for RSW between a 1 mm thick TA2 plate and a 1 mm thick Q235 steel plate. Using a welding current of 7 kA, the resulting joint achieved a tensile shear load of approximately 4.3 kN [11]. Compared with other methods, utilizing Ni foil with an appropriate thickness (e.g., 0.04 mm or 0.06 mm) as the interlayer for the RSW of the TA2 plate and Q235 steel plate enhanced the tensile shear load of the joint. Yu et al. utilized a Cu-Ni alloy as the interlayer, employed a CuCrZr alloy electrode with a diameter of 10 mm on the Ti side and a Cu electrode with a diameter of 8 mm on the steel side, and performed RSW on a 2 mm thick TC4 titanium alloy plate and a 2 mm thick Q235 steel plate, resulting in a joint that achieved a maximum tensile shear load of 12.836 kN [17]. They utilized a Cu-Ni alloy as the interlayer, resulting in a relatively high maximum tensile shear load for the joint when large-diameter electrodes were employed. The following is suggested as a potential approach for the study of the RSW of Ti/steel: within the allowable capacity of the welding equipment, employ electrodes with larger diameters to achieve an increased welding area.

**Figure 8:**
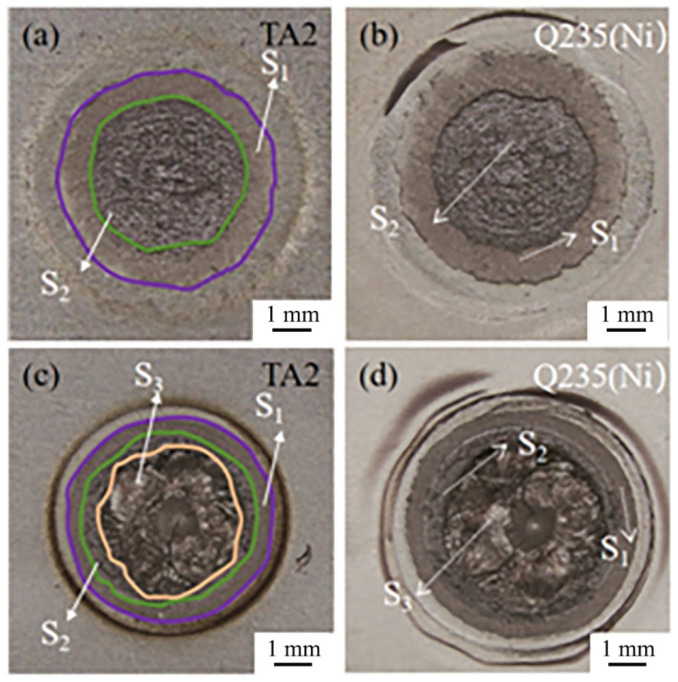
Fractures of Ti/steel joint: (**a**) Ti side of joint containing DS-nugget; (**b**) steel side of joint containing DS-nugget; (**c**) Ti side of joint with C-nugget; and (**d**) steel side of joint with C-nugget.

**Figure 9:**
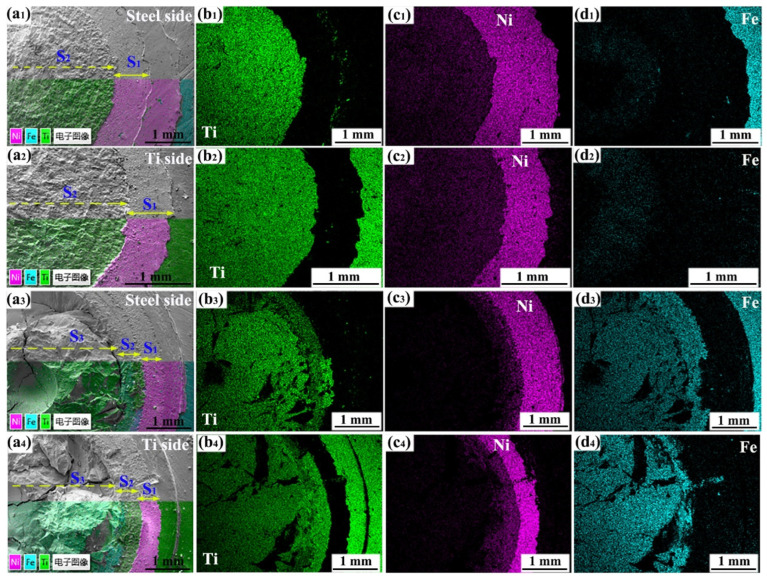
EDS results for the fractures: (**a_1_**–**a_4_**) composite image of SEM- and EDS-layered images for the steel and Ti sides of the joint with DS-nugget and C-nugget; (**b_1_**–**b_4_**) the distribution of Ti on the fractures of steel and Ti sides of the joint with DS-nugget and C-nugget; (**c_1_**–**c_4_**) the distribution of Ni on the fractures of steel and Ti sides of the joint with DS-nugget and C-nugget; and (**d_1_**–**d_4_**) the distribution of Fe on the fractures of steel and Ti sides of the joint with DS-nugget and C-nugget. The Chinese characters in the picture are translated as “electronic image”.

Figure 8 shows the fractures of the Ti/steel joints. Figure 8a,b illustrate the fracture surfaces on the Ti side and steel side of the joint containing the DS-nugget, respectively. This joint was produced under the following welding conditions: a welding current of 7 kA, a welding time of 200 ms, an electrode pressure of 3 kN, and an interlayer thickness of 0.04 mm. According to their morphological characteristics, macroscopically, fractures can be categorized into two distinct regions: the annular region located at the periphery of the fracture and the circular region situated at the center of the fracture. Figure 9a_1_–d_1_,a_2_–d_2_ present the EDS analysis results obtained from the local regions of the steel side and Ti side fracture surfaces for the Ti/steel joint containing the DS-nugget, respectively. As shown, Ni was predominantly detected in the annular region on the fracture surfaces of both the steel side and the Ti side. The annular region is designated as the S_1_ region. In circular regions, Ti and Ni were identified on both the steel and Ti sides of the fracture surfaces. The circular region is defined as the S_2_ region.

Figure 8c,d show the fracture surfaces on the Ti side and steel side of the joint with a C-nugget, respectively. This joint was produced under the welding conditions of the 9 kA welding current and a 0.04 mm thick interlayer. In this case, the fracture can be categorized into three distinct regions: the annular region located on the periphery of the fracture, the circular region situated at the center of the fracture, and the middle circular region positioned between the two. Figure 9a_3_–d_3_,a_4_–d_4_ present the EDS analysis results obtained from the local regions of the steel side and Ti side fracture surfaces for the Ti/steel joint with a C-nugget, respectively. Similarly, Ni was predominantly detected in the annular regions of both sides of the steel and Ti, which was also designated as the S_1_ region. On both the steel and Ti sides of the fracture surfaces, Ti and Ni were identified in the middle circular region, which was also defined as the S_2_ region. In the circular region on the steel and Ti sides of the fracture surfaces of the joint with a C-nugget, both Ti and Fe elements were identified. The circular region is defined as the S_3_ region.

**Figure 10:**
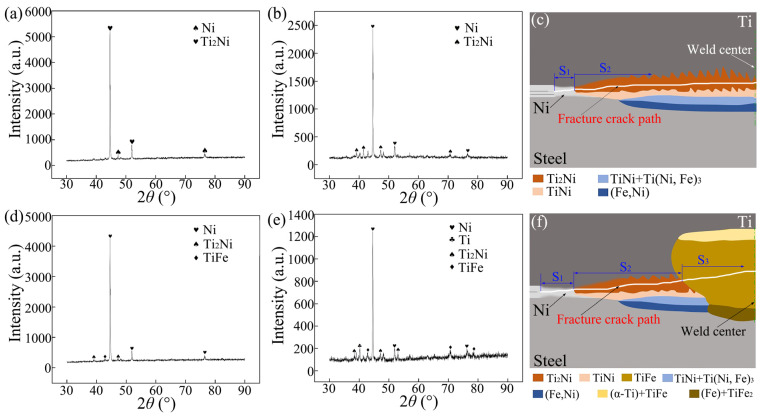
XRD results for the fractures and a schematic diagram of the fracture crack path, (**a**,**b**) XRD results on the steel and Ti side fractures of the joint containing the DS-nugget, respectively; (**c**) a schematic diagram of the fracture crack path in the joint containing the DS-nugget; (**d**,**e**) XRD results on the steel and Ti side fractures of the joint with the C-nugget, respectively; and (**f**) a schematic diagram of the fracture crack path in the joint with the C-nugget.

Figure 10a,b present the XRD analysis results obtained from the fracture surfaces on the steel side and the Ti side of the joint containing the DS-nugget, respectively. Ni and Ti_2_Ni phases were observed on the fracture surfaces of both sides. Based on the EDS analysis results, XRD detection findings, and the morphology of the fracture surfaces on both sides of the joint, together with microstructural observations on the joint cross-section, the schematic diagram illustrating the fracture crack propagation path in the joint containing the DS-nugget during the tensile shear test is presented in Figure 10c. As depicted, the fracture crack initiated between the nickel layers and subsequently propagated within the Ni interlayer. Upon reaching the weld center zone, the crack continued its expansion within the Ti_2_Ni layer. Under external loading, the fracture of the joint is initiated at the unwelded region outside the weld zone. As shown in Figure 3b, the joining between the Ni layer and the Ni layer, as well as between Ni and steel and between Ni and Ti, was well-established around the periphery of the welding zone. However, due to its location at the periphery of the welding zone, the peak heating temperature was relatively low, and the duration of the high temperature was brief [20]. Consequently, little IMCs were formed at the interfaces. In this case, the fracture crack extended within the interlayer of Ni. This crack path corresponds to the S_1_ region, as indicated in Figure 10c. When the fracture crack extended to the welding center zone, it propagated through the more brittle Ti_2_Ni layer, and this region corresponds to S_2_, as shown in Figure 10c.

Figure 10d,e present the XRD analysis results obtained from the fracture surfaces on the steel side and the Ti side of the joint with the C-nugget, respectively. Ni, Ti_2_Ni, and TiFe were identified on the fracture surface of the steel side, while Ni, Ti, and the compounds Ti_2_Ni and TiFe were also detected on the titanium fracture surface. Similarly, the schematic diagram of the fracture crack propagation path in the joint with the C-nugget during the tensile shear test was established, as depicted in Figure 10f. For the Ti/steel joint with the C-nugget, the initiation of the fracture crack and its propagation at the edge of the welding zone are comparable to those observed in the Ti/steel joint containing the DS-nugget. When the fracture crack extended into the mixed nugget, it continued to propagate through the more brittle TiFe layer, as shown in Figure 10f. The failure of the Ti/steel joint occurred within the Ni interlayer, the Ti_2_Ni layer, and the TiFe layer, corresponding to regions S_1_, S_2_, and S_3_, respectively, as indicated in Figure 10f.

**Figure 11:**
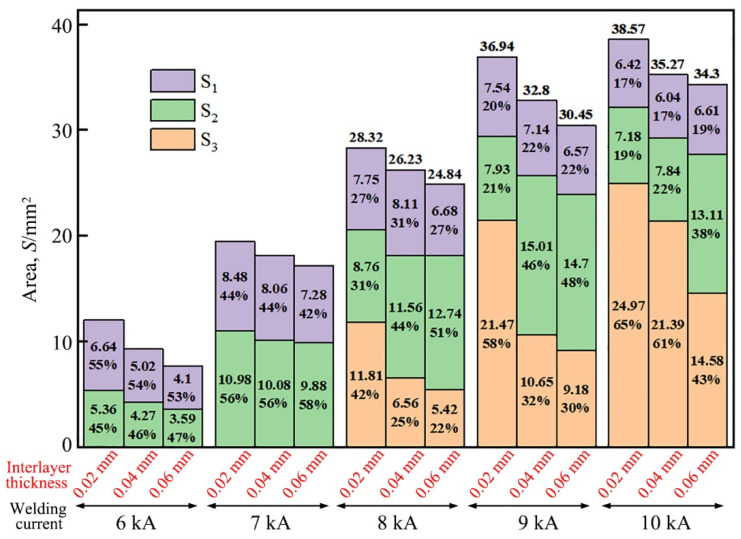
The area of each region and its proportion.

The area of each region was quantitatively measured on the fracture images of the Ti/steel joint. Figure 11 illustrates the measured areas of the S_1_, S_2_, and S_3_ regions on the fracture surfaces of each joint, along with their respective proportions. Although the S_1_, S_2_, and S_3_ regions of the joint may contain small amounts of other phases in addition to their primary constituents—Ni, Ti_2_Ni, and TiFe, respectively—in order to provide a rough estimation of each region’s contribution to the tensile shear load of the Ti/steel joint, it was assumed that the stresses within these regions remained constant across all joints, denoted by σ_1_, σ_2_, and σ_3_, respectively. The values of σ_1_, σ_2_, and σ_3_ were calculated using the least square method as described below [20].

(1) $$S=\sum_{i=1}^{15}r_{i}^{2}=\sum_{i=1}^{15}[P_{i}-(\sigma_{1}A_{1i}+\sigma_{2}A_{2i}+\sigma_{3}A_{3i}){]}^{2}$$

Here, i denotes the index number of the joint; P_i_ represents the tensile shear load of the ith joint; and A_1i_, A_2i_, and A_3i_ represent the areas of S_1_, S_2_, and S_3_ for the ith joint, respectively. The condition for S to attain its minimum value is given by the following equations.

(2) $$\frac{\partial (S)}{\partial (\sigma_{1})}=-2\sum_{i=1}^{15}[{(P}_{i}-\sigma_{1}A_{1i}-\sigma_{2}A_{2i}-\sigma_{3}A_{3i})A_{1i}]=0$$

(3) $$\frac{\partial (S)}{\partial (\sigma_{2})}=-2\sum_{i=1}^{15}[{(P}_{i}-\sigma_{1}A_{1i}-\sigma_{2}A_{2i}-\sigma_{3}A_{3i})A_{2i}]=0$$

(4) $$\frac{\partial (S)}{\partial (\sigma_{3})}=-2\sum_{i=1}^{15}[{(P}_{i}-\sigma_{1}A_{1i}-\sigma_{2}A_{2i}-\sigma_{3}A_{3i})A_{3i}]=0$$

The values of σ_1_, σ_2_, and σ_3_ were calculated to be approximately 364.89 MPa, 275.84 MPa, and 33.63 MPa, respectively, by solving Equations (2)–(4) using the experimental data of the tensile shear load of the joint and the area of each region on the fractures of the joint.

As described above, the S_1_ and S_2_ regions, which mainly consisted of Ni and Ti_2_Ni phases, respectively, played a more significant role in contributing to the tensile shear load of the Ti/steel joint. In contrast, the S_3_ region, primarily composed of TiFe, contributed less to the tensile shear load of the Ti/steel joint. This indicates that when using a Ni interlayer for the RSW of titanium and steel, with appropriately selected welding parameters, only Ti-Ni series IMCs form in the welding interface region, while the formation of Ti-Fe series IMCs was effectively avoided. As a result, the joint performance was significantly enhanced.

## 4. Conclusions

From this study, the following conclusions can be drawn:

When using a Ni interlayer for the RSW of titanium and steel, a distinct nugget forms on each side of the titanium and steel when the welding current is relatively low. When the welding current exceeds 8 kA, the nuggets on the titanium side and the steel side are partially mixed.

In the Ti/steel joint, when the nuggets on both sides of titanium and steel remain unmixed, the IMCs that form in the interfacial zone are predominantly of the Ti-Ni type. Conversely, when the nuggets are mixed, the IMCs formed are primarily of the Ti-Fe type.

As the welding current increases, the tensile shear load of the joint exhibits a trend of initially increasing and subsequently decreasing. When the welding current is 8 kA, the tensile shear load of the joints with an interlayer of 0.04 mm thickness reaches their maximum value, approximately 8.02 kN.

In the Ti/steel joint, the Ni and Ti_2_Ni phases play a more significant role in contributing to the tensile shear load of the joint.

## Figures and Tables

**Table 1:** Chemical compositions of experimental materials (mass fraction,%).

	C	N	H	O	Fe	Mn	P	S	Si	V	Ti
TA2	0.01	0.02	0.002	0.14	0.07	-	-	-	-	-	Bal.
Q235	0.14	-	-	-	Bal.	1.0	0.04	0.02	0.4	0.06	-

**Table 2:** Welding conditions.

	Changing Welding Current			Changing Welding Time		
Interlayer thickness (mm)	0.02	0.04	0.06	0.02	0.04	0.06
Welding current (kA)	6~10	6~10	6~10	8	8	8
Welding time (ms)	200	200	200	100~500	100~500	100~500
Electrode force (kN)	3	3	3	3	3	3

**Table 3:** EDS results for each location marked in Figure 4 (at.%).

	A_1_	B_1_	C_1_	D_1_	E_1_	F_1_	G_1_	A_2_	B_2_	C_2_	D_2_	E_2_	F_2_	G_2_	H_2_
Ti	99.4	76.0	70.5	49.9	49.8	0.6	0.0	99.3	88.1	74.1	70.4	45.9	33.1	4.3	0.0
Fe	0.0	0.0	0.0	0.0	0.0	3.7	99.8	0.0	0.0	0.0	0.0	2.7	17.1	83.1	100
Ni	0.6	24.0	29.5	50.1	50.2	95.7	0.2	0.7	11.9	25.9	29.6	51.4	49.8	12.6	0.0

**Table 4:** EDS results of each location marked in Figure 5 (at.%).

	A_3_	B_3_	C_3_	D_3_	E_3_	A_4_	B_4_	C_4_	D_4_	E_4_	F_4_	G_4_	H_4_
Ti	100	73.6	49.4	46.4	0.2	100	67.9	55.2	52.9	47.1	66.7	21.9	0.0
Fe	0.0	11.2	18.1	9.3	99.8	0.0	29.9	42.1	43.7	48.7	32.6	77.5	100
Ni	0.0	15.2	32.5	44.3	0.0	0.0	2.9	2.7	3.4	4.2	0.7	0.6	0.0

## Data Availability

The data are contained within the article.
